# Barriers to mental health service utilisation in Sudan - perspectives of carers and psychiatrists

**DOI:** 10.1186/s12913-016-1280-2

**Published:** 2016-01-27

**Authors:** Sara H. Ali, Vincent I. O. Agyapong

**Affiliations:** 1 Department of Psychiatry, St Patrick’s University Hospital, Dublin, Ireland; 2 Centre for Global Health, University of Dublin, Trinity College, Dublin, Ireland; 3 Department of Psychiatry, Faculty of Medicine and Dentistry, University of Alberta, Edmonton, Canada; 4 Department of Behavioural Sciences, Kwame Nkrumah University of Science and Technology, Kumasi, Ghana

**Keywords:** Barriers, Mental health, Utilization, Sudan

## Abstract

**Background:**

In order to scale up mental health care nationally, barriers to health services utilisation need to be identified and addressed.

Aim: The aim of this study was to identify barriers to mental health services utilization in Sudan from the perspectives of carer’s of mentally ill patients and psychiatrists and to make recommendations to address the identified barriers.

**Methods:**

Mixed research methods were used in this cross sectional survey. The quantitative part was conducted with carers of mentally ill patients who were staying in Tijani Elmahi psychiatric hospital in Sudan, and the qualitative part was conducted with the psychiatric consultants in the country.

**Results:**

103 carers and six psychiatric consultants participated in the study. According to carers, the main barriers to utilisation of mental health services includes: the beliefs around mental illness, resorting to alternative treatments such as religious and traditional healers, centralization of mental health services, inadequate number of mental health staff, and mental health not being a priority by policy makers. In addition to these barriers, the psychiatric consultants identified stigma, cost of medications, and worries about medication’s side effects as barriers to the utilisation of mental health services. The carers and psychiatrists proposed several solutions to address the barriers to health services utilisation.

**Conclusion:**

Carers and psychiatrists are aware of the barriers to mental health services utilisation in Sudan. Addressing these barriers require a health policy and political response.

**Electronic supplementary material:**

The online version of this article (doi:10.1186/s12913-016-1280-2) contains supplementary material, which is available to authorized users.

## Background

The World Health Organization WHO [1] defines mental health as a state of wellbeing in which individuals realize their own abilities, can cope with normal stresses of life, can work productively, and are able to make contributions to their own community. Mental health is an integral component of health including different aspects of activities which are directly and indirectly related to promotion of wellbeing, prevention of mental disorders, and treatment as well as rehabilitation of people with mental illnesses [2]. According to the WHO mental health atlas 2011–2012, only 36 % of the people in low-income countries have mental health legislations which suggests there is the need to improve mental health and reinforce mental health systems, especially in low-income countries [3]. Despite the recent worldwide focus on mental health, the burden of mental disorders is still on the increase with a projection for depression alone to be the second cause of disability in 2020. Furthermore, about three quarters of those with mental disorders, mainly in low-income countries, have no access to treatment [4].

Several studies suggest the existence of barriers to access and the utilization of mental health services especially in low and middle income countries. In a qualitative survey of international mental health experts and leaders to review barriers to mental health service development, it was reported that the prevailing public-health priority agenda and its effect on funding; the complexity of and resistance to decentralisation of mental health services; challenges to implementation of mental health care in primary-care settings; the low numbers and few types of workers who are trained and supervised in mental health care; and the frequent scarcity of public-health perspectives in mental health leadership represented significant barriers that need to be addressed by national governments. The authors conclude that population-wide progress in access to humane mental health care will depend on substantially more attention to politics, leadership, planning, advocacy, and participation [5] On the bright side, a recent systematic review of literature and a survey of key national stakeholders in mental health identified a large number of programmes which suggest that successful strategies can be adopted to overcome barriers to scaling up mental health services nationally. These barriers include the low priority accorded to mental health, scarcity of human and financial resources, and difficulties in changing poorly organised services [6]. A study in the Niger delta of Nigeria examined the barriers to the utilization of mental health services from the service users’ perspective. The study result revealed there are economic, physical, and cultural barriers to the utilization of mental health services including stigma, poor knowledge of mental health services, centralization of mental services, and waiting time. The research also assessed user’s socio-demographics barriers which could hinder access to mental health services [7]. In South Africa, structural and attitudinal barriers to mental health care were examined together with the predictors of treatment dropout using face to face interviews. Attitudinal barriers, including the lack of perceived need for treatment, stigma, and the perception of mental disorders as a personal weakness were more commonly reported by interviewees than structural barriers such as financial cost and lack of availability of services [8]. Furthermore, inadequate financial and human resources, lack of collaboration and consultation, and not being a priority by policy makers were recognized as barriers to mental health policy implementation in Ghana [9]. Data from Sudan is very limited, with only one study [10] exploring the barriers to the utilization of mental health services in the capital Khartoum from the perspectives of health care providers and policy makers. In this study, the amount of funding, distribution and allocation of mental health services, poor health education, the long duration of treatment, and fear of stigma were identified as barriers to the utilization of mental health services in Sudan. To our knowledge, no study in Sudan has examined the barriers to the utilization of mental health services from the perspectives of carers of mental patients and this study in part seeks to address this gap in the literature.

Given this background, the aim of this study is to identify barriers to mental health services utilization in Sudan from the point of view of both carers of mentally ill patients and psychiatrists and to elicit solutions to address these barriers, thereby expanding mental health services utilization in Sudan.

## Methods

### Conceptual framework

Health Belief Model (HBM) was used to inform the study. This model explains why people fail to engage in certain preventative health behaviours. Conceptualizing mental health care utilization using HBM as a framework has been previously described in an article which proposes that HBM could be used for research in mental health service utilization as well as to communicate to mental health policy makers the need to implement and evaluate effective programs that decrease barriers to treatments [11]. The HBM assumes that behavior change occurs with the existence of three ideas at the same time:

- An individual recognizes that there are enough reasons to make a health concern or concerns relevant (perceived susceptibility and severity).
- That individual understands he or she may be susceptible to a disease or negative health outcome (perceived threat).
- The individual recognises that behavior change can be beneficial and the benefits of that change will be greater than any costs of the behaviour change (perceived benefits and barriers).

For this study, the beliefs of carers of mental health patients rather than the beliefs of the patients themselves are examined. This is because it is more common in Africa for family members to have the burden of seeking mental health care for sick relatives rather than the patients seeking care themselves [12–15]. We extend the model by also examining the perceptions of psychiatrists about what they perceive to be barriers hindering health services utilization by patients and their carers.

### Study design

The study was a cross sectional survey of carers of mentally ill patients and psychiatrists. Mixed qualitative and quantitative methods were used for data collection.

### Study setting

The study was conducted in Khartoum, the capital of Sudan between the period of April and July 2014. Tijani Elmahi Psychiatric Hospital in Omdurman, Khartoum was purposively selected to conduct the study, for both the quantitative and the qualitative studies as it represents the oldest and the largest psychiatric hospital in Sudan that serves patients with different socio-demographic characteristics and backgrounds coming from all parts of the country [16]. Two psychiatric consultants from the Abdalal Alidrisi psychiatric hospital in Khartoum Bahri, Khartoum were purposively selected to participate in the qualitative interview to increase the sample size and get a broader view of psychiatric consultants working in Sudan.

### Data collection

The study protocol was approved by the Ethics and Research Committee of the University of Dublin, Trinity College, Dublin and the Research Ethics Committee of the Ministry of Health, Sudan. Quantitative data was obtained from a cross sectional survey of carers of mental patients who were on admission at the Elijani Elmahi psychiatric hospital. A review of the literature did not identify any published questionnaire which address our study objectives or could aid our data collection. A survey questionnaire for the quantitative study was therefore self-developed by the researchers based on the available literature on the barriers to mental health services utilization from studies in Sudan and other low and middle income countries [7–10] as well as the study objectives. The questionnaire was reviewed by other researchers at the Centre for Global Health, University of Dublin, Trinity College. The questionnaire was translated into Arabic language by the lead researcher before being pre-tested on five carer respondents in Sudan who were not included in the study sample to get an appropriate feedback. The questionnaire was revised based on feedback from the pre-test before being administered to the study participants after providing them with an information leaflet and verbal explanation of the objectives of the study and obtaining their written consent. The final questionnaire contained 31 questions (Additional file 1) which were self-completed by about half of respondents and in the case of the other half, at their request, the lead researcher read out the questions and optional responses to the respondents for them to indicate their responses. It generally took 10-15 min for each respondent to complete the questionnaire. The qualitative interview consisted of three questions regarding the barriers to mental health services in Sudan are and what their proposed solutions to those barriers are. The interview questions were constructed from key issues identified in the literature [7–10] and they were piloted before conducting the study so as to receive an appropriate feedback. The three questions included:

- What do you think about mental health services in Sudan?
- What are the most important obstacles facing people who are seeking mental health services?
- In your opinion, how can these barriers be overcome?

As English is the second Language in Sudan, some doctors preferred to conduct the interview in English, others preferred to speak in Arabic, and others mixed the two languages. All interviews were conducted by the first author (SA) and were audio taped.

### Study participants

Total population list was used to recruit participants for the quantitative study to avoid selection bias and to attempt to include the views of all the carers. This comprised 115 persons documented on hospital records as carers of all the 115 patients admitted to the psychiatric hospital at the time of data collection. No financial or other incentive was offered to the respondents. In addition to the survey, six psychiatric consultants were interviewed by the lead researcher to identify the barriers to mental health services utilization from their perspectives owing to their perceived greater understanding of the problem. The qualitative study participants were purposively selected till data saturation was reached. Of the 115 carer’s of mental health patients on admission who were approached with information leaflets about the study, 103 provided written consent and participated in the study, giving a response rate of 90 % for the quantitative part of the study. All six consultant psychiatrists who were approached agreed to participate in the study giving a 100 % response rate for psychiatrists.

### Data analysis

Computer aided programs (SPSS version 21) and Excel (Microsoft 2008) were used to analyse the quantitative data. Descriptive analysis was done in the form of frequencies and charts. Chi square test was used to compare some demographic variables and the significant differences in some responses. For the qualitative interviews, all recorded data was transcribed and then subsequently translated (in case of Arabic interviews). All transcriptions were read carefully and thoroughly and then thematic analysis was done manually in order to regenerate meaning and structure to the data collected. Different themes were identified from transcripts and similar phrases and words that commonly occurred in the transcripts were categorized under the heading of one theme.

## Results

### Demographics of the carer respondents

Of the 103 respondents, 48 (47 %) were males and 55 (53 %) were females. In all, 75 (73 %) participants were married or cohabiting while the other 27 participants were not married including single, divorced, widowed participants. About 26 (25.2 %) respondents were between the ages of 20 and 30 years,19 (18.4 %) respondents were between the ages of 30 and 40 years, and 58 (56.3 %) were over 40 years. The majority of participants had no education (*n* = 37, 36 %) or primary education (35, 34 %) with the rest having secondary education (21, 20 %) or university and post-graduate education (10, 10 %).

Furthermore, 66 (64 %) carer participants were employed (half of them were manual workers) compared to 37 (36 %) who were not employed including 5 students and 10 retired participants. Moreover, only 29 respondents (28.2 %) earned more than 1000 Sudanese pounds (163 U.S Dollars) per month. With regards to the home of origin, 43 % (*n* = 44) were from central Sudan and Blue Nile area, 37 % (*n* = 38) were from western part of the country, and 20 % (*n* = 21) were from Northern and eastern Sudan. When the carers were asked about which mental health diagnosis their relatives presented with to the hospital, approximately half of the participants (50, 48.5 %) responded that they were uncertain, 27 (26 %) indicated their relative had a psychotic disorder, 15 (14.5 %) said depression, 6 (6 %) said anxiety and 5 (4.5 %) indicated that their relatives suffered from other disorders.

### Organization and distribution of mental health services

About half of carer respondents (51 %) resided in Khartoum while the other half (49 %) resided elsewhere across the country (including Darfur state, Kordufan, Elgadarif, and Eldamazeen) and they just came to the capital Khartoum to seek treatment for their sick patients. Less than half of participants (*n* = 40) reported that the time taken by car to visit the nearest Psychiatric Centre to their residence is less than an hour. Overall 82.5 % of carer respondents who reported that the journey from their residence to the nearest psychiatric health centre was less than an hour were residing in Khartoum compared to only 17.5 % who were residing elsewhere across the country. About a fifth (*n* = 21) said the journey from their home to the nearest psychiatric health centre is more than three hours, 41 (40 %) of them lived between one and three hour from the hospital and a similar number 40 (39 %) said they live less than an hour from the hospital.

In respect of the psychiatrists’ respondents, they identified that there are few psychiatric facilities in the districts and also the psychiatric hospitals are not situated according to catchment area which hinders access to psychiatric services for some Sudanese. Interviewee 1 expressed that: “*if we are taking about primary health, still we are lacking the community psychiatry and primary mental health care so most people come to secondary care and to the hospitals. This limits the resources and facilities to provide the best care for a huge number of patients most of whom could have been treated in a primary care”*. Interviewee 3 also stated: “*I think mental health services in Sudan are not sufficient in terms of its numbers, the number of beds, and number of health cadres……..the services are centralized in Khartoum, other districts have a few facilities”.* This interviewee also illustrated that, for a patient to come from Kordufan state in western Sudan for example, he needs about 2 days travel which makes it unwise to ask him to come every two weeks or even monthly. Interviewee 4 also stated: “*The geography of mental health services is not spread according to the catchment areas. The health services are geographically distant from the populated areas”.*

In respect of solutions to the problem posed by the proximity of health services, one of the doctors suggested mobilization of services in rural areas. Another doctor suggested that; psychiatric hospitals should be more specialized and there should be the presence of psychiatric departments in regional and district hospitals.

### Seeking alternate treatments and beliefs about medication as barriers

The majority of carer participants (*n* = 73) said that their patient’s sought other types of treatments before coming to the psychiatric hospital. Overall, 80 % of respondents who had only primary or no education resorted to other types of treatments before coming to the psychiatric hospital compared to 62.2 % of those with higher education (secondary, university, and post graduate studies). Nevertheless, this difference in response by educational level was not statistically significant (*P* value = 0.06). Consulting religious healers was the main alternative to health care with 66 participants reporting that their sick family members consulted religious healers. When the psychiatrists were interviewed, a theme emerged related to the beliefs and understanding of mental illness. For instance, interviewee 1 stated that; *“Too many people still believe that mental illness should be treated by traditional healers, by religious healers, and other agencies other than doctors”.* Interviewee 2 stated: *“Society is not well aware of the importance of mental health services. They think whatever happens is related to the devil and witchcraft”.* Interviewee 4 also stated: *“Up till today I encounter very few people who came straight to the doctor, usually they have passed through many religious healers and traditional healers before coming here”.* Finally, interviewee 6 stated: *“there are differences in the beliefs about the aetiology of mental illness and who should provide the treatment; the psychiatric doctor or the traditional healer?”*. As a solution to this problem, all the psychiatrists proposed the need to increase peoples’ awareness about the causes and treatments of mental illnesses.

The overwhelming majority of carer respondents (*n* = 95) thought psychiatric medications are very effective, effective, and somehow effective in treating mental conditions including 62 % of respondents with primary or no education who reported that medications are very effective or effective compared to 65.6 % of respondents who had at least secondary level education (*P* value = 0.79). Furthermore, just under half of career respondents (46 %, *n* = 47) reported they do not worry about the side effects of psychiatric medications and about a quarter of the carer respondents (24 %, *n* = 25) reported that they are concerned about the side effects of medication including deterioration in the patient’s mental condition or the medication causing other physical problems. In contrast, psychiatrists proposed medications as a barrier to mental healthcare under the theme of belief and understanding. In this regard, interviewee 1 stated, “*one barrier is the belief around medications. People believe this medication can lead to addiction, to side effects, deterioration of the mental condition, and some believe once you start taking them you will not stop”.* He proposed ongoing public education about the benefits of medication in treating mental illness.

### Stigma as a barrier

Approximately 17 % (*n* = 18) of carer respondents were very concerned and 39 % (*n* = 40) were concerned to some extent about stigmatization of the family members after they were admitted to the psychiatric hospital, whilst the remaining 36 % (*n* = 37) were not concerned. Although, the fear of stigma was not recognized as a reason for delaying or not seeking mental health care by 85 % of carer respondents, statistically significant difference in responses were given by residency, as 23 % of respondents who resided in Khartoum reported that the stigma was a cause of delay in seeking mental health care compared to only 6.4 % of respondents who resided elsewhere across the country (*P* value = 0.02). Of those who reported a delay in seeking health care due to fear of stigma, concerns about other people’s criticisms, patient’s position at work, and patient’s social relationships were cited as barriers to accessing mental health services. When the views of the psychiatrists were sought, one interviewee expressed that: *“Some people still have the stigma of attaching mental illness to their family, so they either refuse to bring the patient to the hospital so that no one will know they have a mental patient”.* This interviewee continued: *‘’to avoid the stigma actually they take patients to traditional healer which is accepted in the community”.* Increasing people’s awareness was also identified as a means to tackle this problem of stigma attached to mental illnesses affecting the treatment seeking behaviour. Another interviewee suggested that the name psychiatric should be removed from mental hospitals as a way of reducing the stigma associated with such facilities. He illustrated this with an example that more patients accepted to come to the hospital after one psychiatric hospital’s name was changed from rehabilitation centre to Abdalal Alidrisi Hospital. On the contrary, one interviewee reported that: “*If you bring your son or relative to hospital like Tijani Elmahi hospital, you know the name of Tijani Elmahi is linked to people with certain sicknesses and he will be labeled as mad. Later he will find difficulties in his personal relations and in marriage. Even his sisters will also be stigmatized and won’t get married as they have a mad brother”.* Another interviewee stressed the importance of having psychiatric departments in general hospitals as a way of fighting stigma: *“Yes we say psychiatric hospitals are important, but the presence of psychiatric departments in general hospitals is important too. This is especially of particular importance for people who are worried about stigma attached to a mentally ill patient, so, if they were asked, they would say that the patient was admitted to Khartoum Hospital without mentioning the department”*. Public education and increasing awareness about mental illness was proposed by some psychiatrists as a way of overcoming the stigma that hinders some people from accessing mental health services. In this regard, one interviewee stated: “*Stigma requires awareness programs using T.V, radio, newspapers, or directly contacting people. Volunteers who had a previous mental illness could share their experiences. We need people to speak in schools and universities to increase people awareness. Also, if doctors, even in other specialties, had an awareness role this will help to improve the situation”.*

### Finance as a barrier

When carer participants were asked about the difficulties in taking care of patients in a psychiatric hospital, 60 % (*n* = 62) reported that they had financial difficulties. Moreover, nearly an equal percentage 57 % (*n* = 59) stated they have to stop their work to stay with the patient in the hospital. Interestingly, about 79 % of carer respondents (*n* = 81) reported the cost of psychiatric medications will not act as a barrier to patients stopping their medication. Overall, 78.5 % of carer respondents who were employed gave such answer compared to 90.9 % of those who were not employed (*P* value = 0.12). Examples of justification to such answers *are: “We have to treat the patient whatever the cost even if we beg for money” “We have to treat the patient even if we stop eating” “We can’t leave the patient without treatment whatever the cost”.* This result differed markedly from what was found from doctor’s perspectives. All the doctors interviewed identified financial problems, specifically the cost of medications, as a challenge facing people and hindering them from accessing mental healthcare. One of the interviewees stated, *“One hindrance is the cost of medication, especially the long term medication”.* These sentiments were expressed by another interviewee who stated: *“The drugs are needed in the long term in most cases and many families can’t meet the financial demands imposed by the cost of medications”.*

In terms of proposed solutions, one of the interviewee’s suggested that psychiatric medications should be free. He said he is an advocate for free psychiatric care including medications. However, he sounded a little pessimistic when he added: “*it is a dream and dreams are free and we can dream as we like”.* Another interviewee suggested the inclusion of psychiatric medications on the health insurance plans when he stated: “*health insurance does not cover psychiatric medications although these drugs are very expensive …I think if they are included, it will decrease the burden on patients and their families”.*

### Health personnel and stakeholders

Overall 48 carer respondents expressed concerns that the number of psychiatrists in the hospital are not sufficient to provide the needed care for the mentally ill in the hospital. All respondents reported that their relatives on admission are seen once a week by the psychiatrists. About half of the respondents (*n* = 50) also expressed that the number of nurses in the hospital are insufficient. Similarly, nearly about three quarters (71 %) mentioned their relatives were not seen at all by social workers, 92 % reported they were not seen by a psychologist or counselor, and about 95 % reported apart from the psychiatrists and nurses, there were no other health personnel in the hospital who came to see the patient including occupational therapist, nutritionist, and pharmacist.

When the psychiatrists were interviewed, they also identified the number of mental health personnel as insufficient. Another psychiatrist inferred that psychiatric education in Sudan is superficial and he suggested an improvement in the undergraduate psychiatric curriculum for medical students in addition to regular postgraduate courses for doctors to improve knowledge and performance in mental healthcare. The psychiatrists also identified poor staff motivation and migration of mental health staff to other countries as barriers to the provision of mental health care in Sudan. One of the interviewees suggested that as a result, mobilization of mental health services in rural areas, establishment of psychiatric departments in general hospitals, and the provision of incentives for doctor’s to accept postings to rural areas as possible solutions. *“If we are talking about primary health, still we are lacking the community psychiatry and introduction of primary mental health care so most people would come to the secondary care, to the hospitals, that makes the resources and facilities to provide the best care for huge number of patients”*.

Nearly all carer respondents answered ‘No’ (95 %, *n* = 96) when they were asked if they thought the government of Sudan allocates enough money for mental health. This result from patient’s carers’ perspective was consistent with healthcare providers’ views. Both groups mentioned that mental health is not a priority with one psychiatrist suggesting that the budget allocated to mental health is almost zero. Another of the psychiatrists stated: *“Mental health has been discriminated by policy makers and it has never been a priority in the health services”.* Consequently, the psychiatrists suggested that mental healthcare should be given the same priority as is given to physical healthcare, with one of the psychiatrists stating: “*If policy makers give the same priority they give to physical healthcare to mental healthcare, then things will improve”.*

## Discussion

Our study reveals a number of barriers to the utilization of mental health services in Sudan from the perspectives of carers and psychiatrists, including physical barriers, attitudinal barriers in seeking alternative care to mental health, mental health system infrastructure, and a low priority for mental health in policy making. Most of these barriers are consistent with the HBM and explain why some mentally ill patients do not access treatments or utilize mental health services [11]. Our results suggest a large proportion of patients travel long distances to access mental healthcare which is located primarily in the national capital Khartoum and this may hinder many people from accessing mental health services. This finding is consistent with the results of previous studies in Nigeria which revealed that the long waiting time as well as the long travel distance to access mental health service act as barriers to services utilization, especially for rural residents [7]. Another study showed that depressed patients living 30 to 60 miles away from psychiatric health services were less likely to receive therapy compared to those who were living within shorter distances [17]. The distance patients have to travel to access mental health care are important modifying environmental factors affecting health seeking behaviour in the HBM [11]. The closer health facilities are to where patients reside, the more likely it is that they will seek health care and vice-versa. The only related study in Sudan [10] did not identify distance as an important constrain to accessing mental health care, however the authors reported that policy makers and health care providers who were interviewed were all of the opinion that mental health services in Sudan are below the minimum acceptable international standards. The psychiatrists interviewed in our study agreed that the number of health personnel in Sudan is much below the number needed to provide adequate mental health care and the few psychiatrists working in Sudan lack the appropriate motivation and incentives and this creates barriers to the provision and utilization of mental health services. These findings are consistent with what were reported in the previous study in Sudan [10]. Lack of staff was also identified as a barrier to access and receive mental health care in a study conducted in Eastern Cape, South Africa [18]. About three quarters of patients, regardless of the educational level of their carers, sought other types of treatments before coming to the psychiatric hospital and over half of all the carers thought the origin of psychiatric illness is spiritual in nature. In the HBM, the likelihood that a person will follow a preventive behavior is influenced by their subjective weighing of the costs and benefits of the action. The response to the perceived threat is influenced by information and the balance between the perceived efficacy and cost of alternative courses of action which creates pressure to act, but does not determine how the person will act [11]. Our study suggests that the beliefs and attitude of people in seeking alternatives to mental health care are important barriers to mental health services utilization, in particular, as consulting religious healers was found to be the main alternative to mental health care. Many people in Sudan perceive that mental illnesses are caused by spiritual forces which may inform their decision to seek help from spiritual and traditional healers in the initial instance rather than seek conventional psychiatric care from mental health services, and this health seeking behaviour is consistent with the HBM [11]. These attitudinal barriers to the utilizing mental health services from carer’s perspective was confirmed by the psychiatrists. These results are consistent with the previous study in Sudan in which all health care providers and administrator respondents agreed that psychiatric patients usually come to hospital in the late stages, after religious or traditional healers have failed to cure their sicknesses [10]. In addition, literature from adjacent countries supports our findings. For example, not only were the beliefs around the origin of psychiatric disorders linked to curses and the effects of the devil’s eye in a study in Ethiopia; consulting religious healers was also found to be the preferred remedy by most of participants, leaving mental health care as a last option [19].

A range of barriers to seeking mental health care in low-and middle-income countries has been investigated. Little, however, is known of the barriers to care and help-seeking behaviour among people with posttraumatic stress disorder (PTSD) in low- and middle-income countries. In a population-based study including 977 people aged 18-40 years with posttraumatic stress disorder from the Eastern Cape Province in South Africa, it was reported that the most striking barriers to mental health services utilisation were stigma and a lack of knowledge regarding the nature and treatment of mental illness [20]. Similarly, of the variety of barriers identified for patients with depression in the same province to seeking mental health services, the most significant were related to stigma, lack of knowledge of their own illness and its treatability, as well as financial constraints [21]. Stigma is therefore a major barrier to mental health services utilisation and it is another important modifying environmental factor in the HBM [11]. Although only a small proportion of carers reported stigma as a barrier to the utilisation of health services, carers who were living in Khartoum reported stigma as a barrier to accessing mental health care significantly more than those living outside the capital (23 % compared to only 6.3 % respectively), indicating the need for more awareness programs, particularly in the capital. In contrast to the perspectives of the majority of carers, the fear of stigma was reported by all the psychiatrists as being among the major barriers to the utilization of mental services in Sudan. The difference between carers’ and psychiatrists’ perspective can be attributed to selection or sampling bias as those carers who participated in the survey were those who had come to hospital to seek mental health care for their relatives. The result could differ if carers were selected from their homes for example. Moreover, it is possible the social or desirability bias played a role in producing such a difference. Accordingly, some psychiatrists suggests that carers of mentally ill patients prefer to send their sick relatives to religious healers to avoid labelling the patients; indicating the interaction of social cultural and belief systems in utilizing mental health services. These observations are consistent with what was reported in the previous studies in Sudan [10] and South Africa [18, 20, 21]. Furthermore, in a qualitative study in Nepal, in addition to pragmatic barriers at the health facility level, mental health stigma and certain cultural norms were found to reduce access and demand for services. Respondents in this study perceived the lack of awareness about mental health problems to be a major problem underlying this, even among those with high levels of education or status. They proposed strategies to improve awareness, such as channelling education through trusted and respected community figures, and responding to the need for openness or privacy in educational programmes, depending on the issue at hand [22].

Although more than half of the cares of mentally ill patients experienced a financial problem in accessing mental health care services, carers felt that they were obliged to treat their mentally ill patients. This suggests the existence of a huge financial burden on carers of psychiatric patients as the cost of the cheapest antipsychotic medication, for example, in Sudan is 72 % of the daily minimum wage [23]. Poverty and absence of social grants for mentally ill patient increase the burden of psychiatric disorders leading to poor mental health outcomes [24]. According to the HBM, someone living in poverty would be more threatened by a disease if they could not afford health care [11]. From health care provider’s perspective, financial problems, especially the cost of psychiatric medications, is one of the major barriers to obtaining mental health care. This is particularly so because patients need to pay for their psychiatric medications which usually require long term use. Thus, although financial barriers related to medications were among the major themes which emerged from the study according to the psychiatrists, the cost of psychiatric medications were not identified as hinders to access mental health services by a large proportion of carers. In contrast to carers’ perspective regarding financial barriers in this study, carers and service users in Nigeria experienced financial difficulties in paying for their psychiatric medications and follow-up appointments [7].

Our results also suggest that the majority of carers and some psychiatrists perceive that mental healthcare is not accorded sufficient priority by policy makers in Sudan and the government does not allocate sufficient funding for mental healthcare which creates barriers to the provision and utilization of adequate mental health care services. The previous study in Sudan did not clearly identify policy makers as barriers to the provision and utilization of mental health service [10]. However, the lack of funding from policy makers and low priority for mental healthcare were identified as barriers to mental health policy implementation in Ghana [9]. Furthermore, the problem of poor psychiatric education among medical students and doctors which has been identified in our study as a barrier to mental health services utilization was not reported in the previous study [10]. Nevertheless, the issue of poor psychiatric training in Sudan has been highlighted by the WHO [23] who reported that none of the nurses who graduated in 2008 had up to one year training in mental health care and only 0.05 psychiatrists per 100.000 had up to one year training in mental health care in 2008. Furthermore, the problem of poorly trained staff was identified by health professionals as a quality barrier to access mental health care in Easter Cape, South Africa [18].

Various solutions have been proposed by respondents in our study to tackle the different challenges hindering mental health patients from utilizing mental health services in Sudan. Although all the proposed solutions require good policy coordination for effective implementation, our study suggests that policy makers themselves are a major part of the problem. Consequently, as a start, policy makers need to recognise and understand the different dynamics at play within mental health care delivery system [25]. Psychiatrists, human rights advocates and civil society and not for profit organisations all have roles to play in educating and lobbying policy makers so that they prioritise mental healthcare. Political will was recognized as the major solution to improve the problem together with the advocacy for people with mental illness [5]. Secondly, increasing people’s awareness of mental health through educational programs and using different types of media platforms was recognized as a solution to overcoming stigma and improving mental health services utilization, and these approaches are consistent with those proposed in the previous study in Sudan [10]. In addition, improving public awareness through using pictures, posters, religious idioms, and simple language for communication was suggested as a means of improving mental health services utilization in an article that discussed the experience of programs in different low income settings [26]. Opening of more psychiatric health centres, opening of psychiatric departments in general hospitals and mobilization of mental health services to rural areas were recommended by our study participants. In the previous study in Sudan, policy makers advocated for the integration of mental health services into primary care and other facilities [10]. According to the WHO [23], integration of mental health in general services together with creation and strengthening of community based facilities such as psychiatric outpatient facilities and community based inpatients units can strengthen the mental health system in Sudan. Furthermore, as a solution, mental health staff should be motivated, through provision of attractive working environment, to provide the required level of mental health care. Health professionals need to be encouraged to work in rural areas as there are hospitals without psychiatrists. The WHO also proposed increasing the number of mental health staff as a way of strengthening the mental health system in Sudan [23]. According to [27], community participation and working in relationships between different local services or collaboration were found to be effective in improving health services utilisation. Task-shifting of responsibilities from psychiatrists to community health workers and community lay counsellors have also been reported to be effective in increasing the mental health workforce in poor countries [28–33]. Sudan can adopt this strategy to increase the skills mix of it mental health work force at the community level and thereby, expand on the mental health coverage for the population. This may also be an effective tool to mitigate the long distances people need to travel to access conventional mental health services. Offering free psychiatric medication to psychiatric patients and their inclusion in the health insurance was recommended by some psychiatrists as a way of increasing mental health services utilization in Sudan. Health insurance coverage as a successful policy to improve patient’s access to psychiatric medications was suggested by policy makers in the previous study in Sudan [10]. Furthermore, increasing the availability of essential psychiatric medications was recommended by the WHO [23] to strength mental health system in Sudan. Finally, improvement in the process of identification, diagnosis, and treatment of mental health conditions has been recommended as a way to overcome barriers to the availability, accessibility, efficiency and equity of mental health care in low and middle income countries [25].

Our study has a number of limitations. First, mental health patients who are the most important stakeholders when considering barriers to mental health services utilisation in considering a health belief model were not included in the study due to ethical concerns as most admitted mental health patients were considered not able to provide a valid consent need for participation in such a study. The authors have however planned a follow-up study with recovered patients as well as spiritual and traditional healers who are also important stakeholders. Second, although the survey questionnaire was researched based on available literature and piloted before use, it is nonetheless not a validated instrument which limits the validity of the study findings. Third, the small sample size for both psychiatrists and careers limited our ability to conduct subgroup analysis on our results. It also limits the generalizability of the study findings. These limitations notwithstanding, our findings provided the first preliminary literature on the factors which hinder mental health services utilization by patients in Sudan from the perspectives of carers of mentally ill patients, which is a major strength of this study.

## Conclusion

Our study has identified barriers to mental health services utilization in Sudan from the perspectives of carer’s of mentally ill patients as well as from the perspectives of psychiatrists. Overall, our findings are consistent with the HBM and also in line with previous research which reports the presence of several barriers to the utilization of mental health services including: people’s beliefs and understanding, cultural, attitudinal, financial and political barriers. These barriers seem to be interacting and leading to each other hindering proper mental health care in Sudan. In addition to increased public education and awareness creation about mental health to reduce stigma, quality mental health services provided by well-trained and well-motivated staff need to be close to where people live to prevent the travel burden. Furthermore, psychiatric medication need to be made free as a way of increasing the utilization of health services. Finally, an expansion in the mental health services delivery and utilization in Sudan require the active support of policy makers who should consider mental health as a priority.

## Additional file

Additional file 1:
**Questionnaire in English: exploring the barriers to utilization of mental health services in sudan.** (DOC 33 kb)

## Abbreviations

WHO: World Health Organisation
HBM: Health Belief Model
SPSS: Statistical Package for Social Sciences
