# Supplementary material for: Barriers to mental health service utilisation in Sudan - perspectives of carers and psychiatrists
Source: BMC Health Serv Res. 2016 Jan 27;16:31. doi: 10.1186/s12913-016-1280-2 (PMC4729143; doi:10.1186/s12913-016-1280-2)
Supplement: Additional file 1: — Questionnaire in English: exploring the barriers to utilization of mental health services in sudan. (DOC 33 kb) [file 12913_2016_1280_MOESM1_ESM.doc]

**APPENDIX 1**

QUESTIONNAIRE IN ENGLISH: EXPLORING THE BARRIERS TO UTILIZATION OF MENTAL HEALTH SERVICES IN SUDAN

1. Participant ID ___
2. Age?
3. Gender?

Male_____ female____

1. Race, ethnicity?

Northern Sudan___

Southern Sudan___

Western Sudan____

Eastern Sudan____

Capital ____

If other please specify_____

1. Educational level?

Primary ___

Secondary____

Tertiary (university, institution, nursing school ….etc) __

Informal education (trade) ___

Non educated__

1. Marital status?

Single—

Married__

Widow__

Divorced__

1. Employment status?

Employed__

Not employed—

Retired___

Student___

1. Socioeconomic status?

High (earn more than 3000 Sudanese ponds monthly) ___

Middle (earned from 1000 to 3000 Sudanese ponds monthly) ___

Low (earned less than 1000 Sudanese pounds monthly) ___

1. Where do you live?

I live in Khartoum___

I came from other state___

If other please specify___

1. What is the approximate travel time from your home to the nearest psychiatric health center by car?

Less than 1 hour ___

1 to 3 hours___

3 to 5 hours___

More than 5 hours __

1. What mental health problem your relative suffers from?

Depression___

Anxiety___

Mania____

Psychosis___

Others____

1. For how long is your relative has a mental problem before seeking a psychiatric health service?

Less than 1 week__

1 week to 1 month__

1 month to 3 months__

3 months to 6 months___

More than 6 months___

1. If it took you more than a week, why it was the case? Tick more than one if applied

I didn’t think this was a mental problem__

The hospital is too far away__

Financial reasons___

Taking relative to the hospital will stigmatize the family__

We tried other treatment first___

1. Did your relative seek any other type of treatment before admission to the hospital?

Yes___

No___

If your answer is yes, what the type of profession was seen?

General Doctor__

Religious healer__

Herbalist___

Zar ____

If other please specify__

1. Before you brought your relative to the hospital, what you belief about the condition he suffers from?

I thought it was spiritual problem___

I thought it was mental problem___

I thought it was physical problem___

I thought it was physical and mental problem___

If other please specify________

1. Now the patient in the hospital, do you still have the same belief?

Yes__

No__

If the answer is No, what you belief to be the problem now?

Mental ____

Physical____

Spiritual ____

If other please specify___

1. What do you think about the treatment used for psychiatric disorders?

Extremely effective___

Effective____

Somewhat effective__

Not effective___

Not sure___

1. Do you have any concern about the side effect of psychiatric treatments?

Extremely concerned___

Concerned____

Somewhat concerned___

Not concerned ___

1. What concern you have about psychiatric treatments?

Could change the personality__

Could affect the physical health__

Could worsen the condition__

If other please specify__________

1. How concerned you about your family being stigmatized?

Extremely concerned__

Concerned___

Somewhat concerned__

Not concerned__

1. If you concerned about stigma, did it affect your decision to bring your relative to the hospital for treatment?

Yes___

No___

If your answer is yes please explain?______________

1. When you brought your relative to the hospital, how quickly he was seen by a psychiatrist?

Immediately___

With some delay___

With significant delay___

Not seen at all___

1. Since you brought your relative to the hospital, how frequently he was seen by psychiatrist?

Daily___

Weekly___

Monthly___

More___

1. Which other professionals have come to see the patient?

Nurse Yes ____ no_____

Social worker yes____ no_____

Occupational therapist yes____ no______

If other please specify________

1. Do you think there is enough psychiatrists for the number of patients who attend this hospital?

Yes____

No____

Please explain your choice of answer_______________

1. Do you think there is enough nurses for the number of patients who attend this hospital?

Yes___

No___

Please explain your choice of answer_________________

1. What challenges you face in looking after your relative in the hospital? Tick more than one if applied

I have no one else to look after my family__

I have no accommodation in the hospital__

I have to give up my work__

I don’t have enough money__

1. Do you think the cost of medications will affect the likelihood that your relative (patient) will continue treatment after discharge?

Yes___

No___

Pease explain your choice of answer________

1. Do you think stigma will affect the patient follow up for treatment?

Yes__

No__

Please explain your choice of answer__________

1. Identify any concern of treating patients in a specialized mental health services?

Confidentiality. yes__ No_

Long waiting list. yes__ No__

The number of health care providers are not adequate to deliver care. yes__ No__

It cost too much money. yes__ No____

The hospital environment is not well equipped to accommodate the patient. yes__ No__

1. Do you think the government is investing sufficient fund in provision of mental health in Sudan?

Yes__

No__
